# Isolated Pneumopericardium: A Rare Manifestation of Penetrating Chest Trauma

**DOI:** 10.7759/cureus.37071

**Published:** 2023-04-03

**Authors:** Ioannis D Passos, Georgios E Papavasileiou, Konstantinos Fortounis, Christos Papavasileiou

**Affiliations:** 1 Surgery, Mobile Army Surgical Hospital, Didymoteichon, GRC; 2 Surgery, Papageorgiou General Hospital of Thessaloniki, Thessaloniki, GRC

**Keywords:** general trauma surgery, penetrating stab wound, tension pneumopericardium, traumatic pneumopericardium, pneumopericardium, penetrating chest injuries, chest trauma

## Abstract

Pneumopericardium (PPC) is a clinical entity defined by the presence of air in the pericardial sac. It occurs mainly in patients who sustain blunt or penetrating chest trauma and may coexist with pneumothorax, hemothorax, rib fractures, and pulmonary contusions. Although it is a strong indicator of cardiac injury and therefore requires immediate attention for possible surgical treatment, it still remains a commonly misdiagnosed condition in the trauma bay. Only a few cases of isolated PPC associated with penetrating chest trauma have been reported to date. We present the case of a 40-year-old man who was stabbed in the anterior chest, specifically in the left subxiphoid area and left forearm. Imaging, which included chest x-ray, chest computed tomography, and cardiac ultrasound, demonstrated the presence of rib fractures in addition to isolated PPC, with no pneumothorax or active bleeding. The patient was managed conservatively and actively monitored for three days and remained hemodynamically stable upon discharge. PPC is an uncommon clinical entity, suggestive of severe thoracic trauma. Clinical features may include chest discomfort and dyspnea, while asymptomatic patients have also been reported. Since it can be monitored by electrocardiograms and cardiac ultrasound, its presence is not an absolute indicator for surgical intervention, while the treatment plan should be based on the patient's clinical indications and symptoms.

## Introduction

Pneumopericardium (PPC) is a rare clinical entity defined by the presence of air in the pericardial sac, particularly affecting patients sustaining blunt or penetrating chest trauma, that requires a high level of clinical suspicion and multidisciplinary approach [1]. The pathophysiology of PPC is similar to air leak syndrome, a clinical condition characterized by the escape of air from an air-containing cavity to areas that normally do not contain air ​[1]​. PPC can lead to tension PPC (tPPC), a very commonly misdiagnosed life-threatening condition that mimics cardiac tamponade and requires urgent surgical intervention. In postmortem and clinical radiology, PPC is considered an indicator of severe cardiac injury, with most cases presenting with a bilateral pneumothorax ​[2]​. We present a case of isolated PPC secondary to penetrating chest trauma that was managed conservatively. The rarity of this phenomenon was supported by a literature review. According to recent data, PPC is no longer considered an absolute indicator of invasive treatment, and thus a conservative management plan is beneficial to the patient [3]. The current case report highlights the significant diagnostic difficulties of PPC in the trauma unit, while early diagnosis and treatment can significantly reduce mortality rates.

## Case presentation

**Figure 1:**
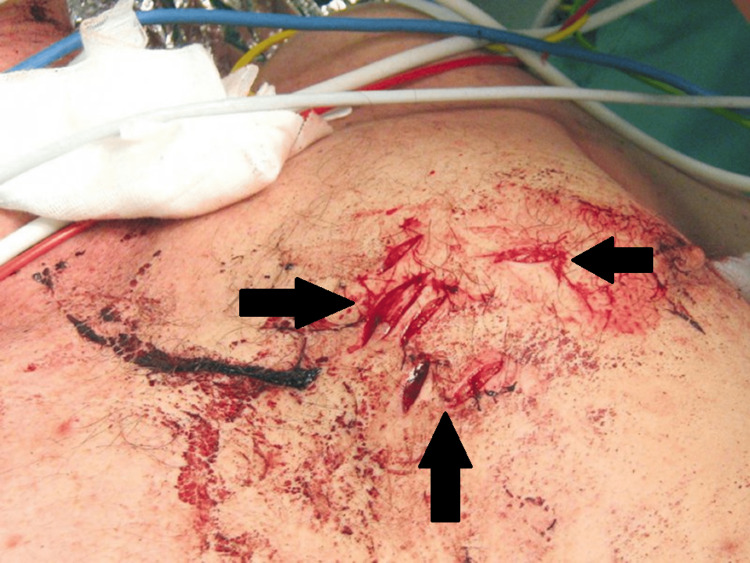
Patient's stab wound of the lower third of the sternum, just parasternally through the left edge of the sternum.

**Figure 2:**
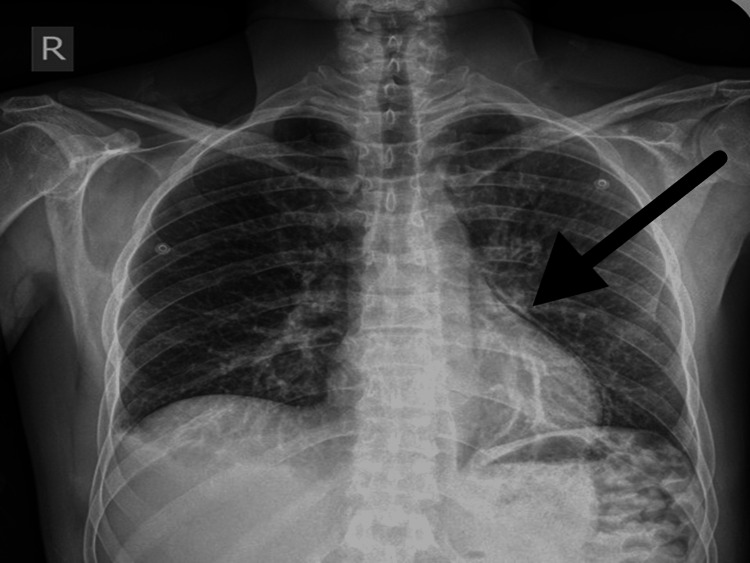
Admission x-ray demonstrating air in the pericardial sac.

**Figure 3:**
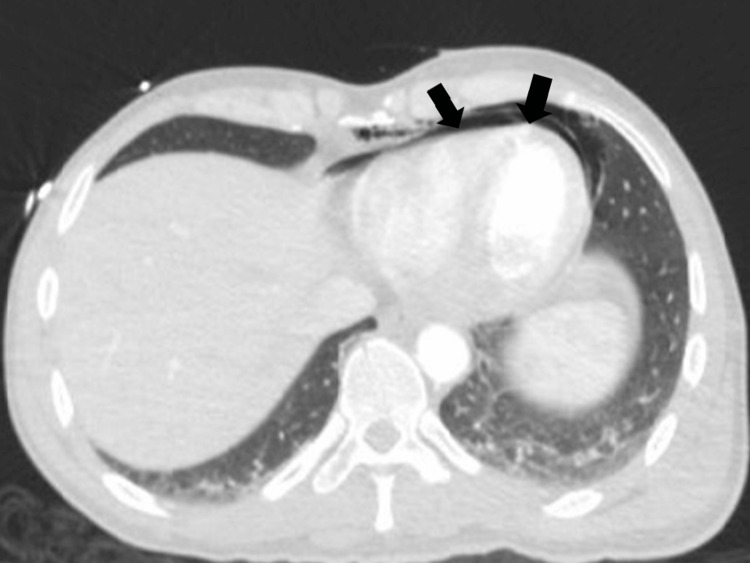
Admission chest computed tomography confirming the pneumopericardium.

A 40-year-old man with an unknown medical history was transported by ambulance to our hospital's emergency department after allegedly being attacked and stabbed in the chest and left forearm 35 minutes earlier during a conflict with compatriots. He sustained penetrating trauma due to a stabbing through the left border of the sternum, and a left forearm laceration with active external bleeding (Figure 1). He was phonating and breathing normally (equal bilateral breath sounds), while his respiratory rate was 22/minute, SpO_2_% was 94%-98%, and the Glasgow Coma Scale (GCS) score was 15/15. He remained hemodynamically stable during the resuscitation and diagnostic workup. A tourniquet was placed proximal to the injured vessel, approximately 3 inches from the nearest wound edge, and a right subclavian central venous catheter was inserted. Laboratory workup revealed slightly elevated markers of cardiac injury: Troponin Ic: 0.17 (normal range < 0.04) and CK-MB: 28 (normal range < 25). Chest computed tomography (CT) demonstrated the presence of chest fractures in addition to an isolated PPC, with no active bleeding from any suspicious region or the presence of a pneumothorax (Figures 2, 3). Cardiac ultrasonography (U/S) showed that the cardiac dimensions were normal, along with the contractility of both ventricles, while there was no pericardial fluid.

CT angiography of the left upper extremity was performed, showing good perfusion of the branchial, radical, and ulnar arteries without any extravasation of the contrast medium. The plastic surgery team operated on the patient's arm injury and sutured the nerve bundle of the dorsal branch of the median nerve without finding any serious vascular injuries. In response to his chest injury, his isolated PPC was managed conservatively with three days of continuous noninvasive cardiopulmonary monitoring and serial cardiac U/S and chest x-rays, which showed slow but steady absorption of the air in the pericardium. He remained hemodynamically stable, having normal breath sounds, oxygen saturation, and ABGs upon his discharge on day 4, he had an uneventful clinical recovery. Regular follow-up in the outpatient clinic seven days later showed no clinical, biochemical, or radiological deterioration.

## Discussion

While the available literature has extensively uncovered cases of spontaneous or blunt impact PPC, there is still limited data on penetrating trauma PPC. The main results of studies on PPC after penetrating chest trauma are summarized in Table 1.

**Table 1:** Studies on PPC following penetrating chest trauma (literature review). Abbreviations: CR: Case Report; CT: Computed Tomography; N/A: Not Available; SPW: Subxiphoid Parasternal Window; tPPC: tension Pneumopericardium; U/S: Ultrasound; VATS: Video Assisted Thoracoscopic Surgery

	Design	No Patients	Status	Pneumo/Hemothorax	tPPC	Other signs	Απεικόνιση	Management	Days of Hospitalization	Mortality
Knottenbelt et.al, 1989 ​[4]​	Retrospective	2	Unstable	1/2	1/2	-	X-ray, U/S	Thoracotomy, Aspiration	3	0%
Demetriades et.al 1990 ​[5]​	Prospective	20	4/20 Unstable	13/20	1/20 36Hr	ECG: 5/20 elevated ST	u/s: 3/20 small effusion	1/20 laparotomy,13/20 chest tube, 1/20 thoracotomy,5.20 Conservative	N/A	0%
Rashid et al, 1999 ​[6]​	CR	1	Stable	No	No	pneumoperitoneum	X-Ray,CT	Laparotomy, chest tube	5	0%
Tansel et al, 2003 ​[7]​	CR	1	Stable	Yes	Yes, 24Hr	-	X-rays, CT	Thoracostomy, SPW	6	0%
Manson et al, 2005 ​[8]​	CR	1	Stable	Yes	Yes	Abdominal injury	X-ray	SPW, Laparotomy	6	0%
Gasparovic et al, 2004 ​[9]​	CR	1	Unstable	Yes	No	Abdominal injuries	Ct	Chest tube, laparotomy	32 d	0%
Sun et al, 2010 ​[10]​	CR	1	Unstable	Yes	yes	-	Xray	Sternotomy,pericardial drain	N/A	0%
Nicol et. al, 2014 ​[11]​	Prospective	27	6/27 unstable	N/A	1/27	2/27 acute abdomen, 2/27 cardiac tamponade, 1/27 tPPC	X-rays, 19 U/S,CCT, CCTA 2/27	19/27 SPW, 2 sternotomies, 3/27 laparotomies	Mean:6.5	0%

The most common mechanisms of air entering the pericardial sac include: i) prolonged positive airway pressure, particularly in the presence of parenchymal pulmonary contusion; ii) direct communication with the external environment; iii) airflow from ruptured alveoli; iv) concomitant lesions of the esophagus or bronchial tree; and v) congenital communication of the pleural and pericardial spaces [12,13]​. Yet if the visceral pleura remains intact, as in our case, the patient may develop isolated PPC, a clinical entity that appears to occur in less than 0.2% of chest trauma patients ​[3,14]​. In cases of blunt chest trauma, PPC can occur as a result of alveolar rupture caused by a sudden rise in intrathoracic pressure, culminating in an air leak into the pericardium space in the presence of a pleuropericardial tear. In the case of visceral pleura or lung interstitium disruption, the patient may develop pneumothorax or air leak into the mediastinum, neck, or retroperitoneum, a phenomenon known as “Macklin effect” ​[15]​. Other proposed mechanisms entail direct air entry into the pericardium via congenital or traumatic pleuro-pericardial contact, or from a force strong enough to rupture the trachea, bronchus, or pericardium ​[15]​. As mentioned above, the air in the pericardium can infrequently compress the heart and cause tPPC, which presents radiographically as a globally small cardiac silhouette and clinical signs of cardiac tamponade ​[14]​. tPPC following blunt trauma is an uncommon condition associated with increased fall height, as well as aortic, pericardial, and myocardial ruptures ​[2]​. 

According to retrospective studies, up to 10% of PPC cases associated with chest trauma develop tPPC within the first 24 hours, with mechanical ventilation correlating with 33% of them [15]. PPC without tension occurs when pericardial pressure rises above the normal range of 50-100 mmHg, while tension PPC occurs when pressure rises above 145 mmHg. The occurrence of air tamponade is of particular concern as the mortality rate from tPPC is approximately 57% [16]. It has been hypothesized that the volume of air required to build tPPC increases as a result of the one-way valve effect combined with increasing pleural pressure caused by positive pressure breathing [13]. tPPC induces hemodynamic instability by restricting cardiac contractility and venous return to the heart, resulting in decreased cardiac output. Clinically, it is the Becks' triad (hypotension, muffled heart sounds and increased jugular venous pressure) as well as tachycardia, chest discomfort, tachypnea and pulsus paradoxus [17]. Bruit de Moulin sign (mill wheel murmur), which is usually accompanied by precordial displacement tympania, is a rarer, more specific symptom, although cardiac tamponade produced by PPC or fluid has identical clinical features [10].

In terms of laboratory testing, cardiac injury markers (i.e., CPK, CK-MB), while they may indicate possible myocardial injury, do not appear to correlate with the occurrence of PPC. Electrocardiogram (ECG) is also non-specific, with bradycardia, low-voltage recordings, pericarditis-like abnormalities, or non-specific ST wave changes observed [5,10]. The diagnosis of PPC and tPPC remains a challenge in the trauma bay. It has been suggested that initial imaging in hemodynamically stable individuals should include an erect chest x-ray, ECG, and U/S scan of the pericardium, since an occult penetrating chest injury can cause rapid deterioration in the patient's condition and be fatal [11]. Focused abdominal ultrasonography for trauma (FAST) in the context of Advanced Trauma Life Support (ATLS) protocol, on the other hand, has been described as unreliable for the diagnosis of PPC, emphasizing the importance of x-rays [16]. However, according to certain reports, the subxiphoid FAST view, used to assess pericardial effusion, can occasionally show a portion of the cardiac image obscured by A-lines that indicate PPC [18]. Since chest trauma patients usually present with concomitant pneumothorax or pneumomediastinum, the CT scan can be crucial to detect PPC and tPPC in hemodynamically stable individuals. Occasionally, normal anatomical features (i.e., large fissure or the anterior line of communication) may resemble air in the mediastinum. In addition, the Mach-band effect, which appears as a zone of lucency surrounding structures with convex edges, can be used to simulate pneumomediastinum. The lack of an opaque line, which is prevalent in pneumomediastinum, can aid in differentiation ​[19]​.

In the case of tPPC, PPC distension and cardiac compression support the diagnosis of tension, while transthoracic echocardiography in conjunction with diagnostic video-assisted thoracoscopy for penetrating injuries appears to be the gold standard for their identification [16,20].

Therapeutically, PPC is not an absolute indicator for surgical intervention, and the treatment plan should be based on the patient's hemodynamic status and clinical signs and symptoms. Individuals with a negative U/S and no hemothorax may be safely discharged [11]. While the presence of PPC in blunt chest trauma is generally considered incidental and benign, there is conflicting evidence in penetrating chest injuries. According to two large prospective studies on the management of PPC in patients with blunt and penetrating chest injuries, a conservative treatment plan has no impact on mortality in stable patients; therefore, sternotomy is not required [3,5]. Therefore, patients with asymptomatic PPC can be closely monitored to avoid escalation to tPPC-associated cardiac tamponade. However, another prospective study of 27 patients concluded that a subxiphoid pericardial window (SPW) should be performed routinely in all individuals with penetrating chest trauma to prevent the development of tPPC, as it has been reported that up to 10% of these patients develop it within the first 36 hours [5,11]. In the event of shock on arrival or an entry wound above the precordium, some authors recommend a more aggressive approach with immediate thoracotomy [5]. Under all circumstances, tPPC is the most likely diagnosis when patients with penetrating chest trauma deteriorate abruptly or develop symptoms of cardiac tamponade with no apparent free fluid in the pericardium. Treatment of tPPC involves early detection and immediate decompression of the pericardial cavity, which should be done prior to mechanical ventilation of the patient, since it carries a high risk of PPC exacerbation due to increased air pressure in the lungs [14]. tPPC can be treated by pericardial decompression using needle pericardiocentesis or percutaneous drainage, followed by soft tubular drain implantation in the operating room, subxiphoid approach, open thoracotomy, or video-assisted thoracoscopy window [10].

## Conclusions

PPC is a rare clinical manifestation of severe blunt or penetrating trauma that can cause chest discomfort and dyspnea, although asymptomatic patients have been documented. Laboratory tests and ECGs are often inconclusive. Its presence in hemodynamically stable individuals is not a clear indicator for surgical intervention, as it can be safely monitored with ECG and cardiac U/S. Therefore, the patient's clinical signs and symptoms should remain the mainstay of the treatment plan. As the mortality rate remains unacceptably high due to frequent misdiagnosis, physicians should be aware of the possibility of tPPC development mimicking cardiac tamponade, since sudden hemodynamic deterioration or clinical symptoms of cardiac compression necessitate immediate decompression.
